# Potential antimicrobial activity, antioxidant, and anti-wrinkle effects of *Linum usitatissimum* oil nanoemulgel

**DOI:** 10.1371/journal.pone.0362294

**Published:** 2026-11-05

**Authors:** Ahmad M. Eid, Mohammed Hawash, Linda Issa, Areej Joma, Ansam Joma, Deema Tumeh

**Affiliations:** 1 Department of Pharmaceutical Chemistry and Technology, Faculty of Pharmacy, An-Najah National University, Nablus, Palestine; 2 Department of Cosmetic and Skin Care, Faculty of Pharmacy, An-Najah National University, Nablus, Palestine; 3 Program of Pharmacy, Faculty of Pharmacy, An-Najah National University, Nablus, Palestine; Strides Pharma Science Limited, UNITED STATES MINOR OUTLYING ISLANDS

## Abstract

**Background:**

*Linum usitatissimum* (*L. usitatissimum*) seeds have been reported to have several biological efficacies, such as antioxidant, anti-aging, sun protection, and antimicrobial actions. The present study aimed to develop *L. usitatissimum* oil nanoemulgel and evaluate its cosmeceutical and antimicrobial characteristics for use in topical applications.

**Method:**

*L. usitatissimum* oil nanoemulgel formulations were prepared by optimizing the non-ionic surfactant (Tween® 20, Tween® 80, and Span® 80) using a self-nanoemulsifying technique, followed by the addition of Carbopol®. The droplet size, polydispersity index, zeta potential, and rheological behavior of the emulgel formulations were measured. In addition, the antioxidant activity, elastase inhibition activity, sun protection factor (SPF), and antimicrobial properties were evaluated.

**Results:**

The prepared nanoemulgel had a homogenous appearance and a smooth, creamy texture and was found to have good spreadability and an acceptable adhesion to the skin. Tween® 80 gave the optimum nanoemulgel formulation, which had a droplet size of less than 200 nm. The crude *L. usitatissimum* seed oil was found to possess strong intrinsic antioxidant activity (IC_50_: 3.15 ± 0.46 µg/ml) when compared to trolox (IC_50_: 2.21 ± 0.23 µg/ml) and elastase inhibition activity (IC_50_: 17.3 ± 0.5 µg/ml) when compared with oleanolic acid (IC_50_: 10.2 ± 0.3 µg/ml), and a sun protection factor (SPF) of 5.87, suggesting significant potential anti-wrinkle and photoprotective properties. When incorporated into the optimized nanoemulgel formulation, the system showed considerably higher antimicrobial activity against *S. aureus*, MRSA, and *C. albicans* than the crude oil (p < 0.05). No antibacterial activity was found against *E. coli*, *E. faecium,* or *S. sonnei*.

**Conclusion:**

In conclusion, a novel *L. usitatissimum* oil nanoemulgel was prepared that shows promise for pharmaceutical and cosmeceutical applications.

## 1. Introduction

Nanotechnology is an essential method for the delivery of pharmacological and cosmeceutical medicines, especially those with poor water solubility [1]. Nano-based delivery systems optimize drug solubility, stability, bioavailability and controlled release and increase patient compliance [2], and nanotechnology can be defined as the employment of science to make extremely small structures using different tools and systems [3]. Pharmaceutical nanodosage forms have special physiochemical and biological properties as compared with their bulk forms [4]. This technology has many advantages in the pharmaceutical industry; it is used to deliver drugs to a specific site, making the drug more effective and cheaper, with fewer adverse effects [5], which leads to an improvement in patient compliance. Furthermore, due to their large surface area, nanodosage forms improve drug absorption, increase passage of poorly soluble drugs into the cell, and improve drug stability. The use of nanodosage forms has ensured great progress in the development of drug delivery systems, some of which are nanoemulsions, self-nanoemulsification, liposomes, nanoemulgels, and polymer micelles [6–9].

Nanoemulsion is a drug delivery system consisting of emulsified oil and water, with an average droplet size of 5 − 200 nm [10]. Nanoemulsion is a stable system, and therefore creaming, flocculation, and aggregation do not occur [11]. It can deliver both hydrophilic and lipophilic drugs [12] and can be administered by various routes such as orally, topically, and parenterally. An emulsion is a system consisting of at least two immiscible liquid phases, one of which is dispersed as globules in the other. It is an important dosage form for poorly water-soluble drugs [13,14]; however, emulsions have some limitations, since their stability may be affected by parameters such as pH and temperature [15]. Furthermore, emulsions can be exposed to creaming, coalescence, and flocculation.

Gel is a transparent semisolid dosage form with good stability and application in comparison with cream and ointment; however, it is inefficient for the delivery of poorly soluble drugs [16]. To overcome these limitations of gels and emulsions, they can be combined in the form of emulgel [17], which has a high solubilizing ability and permeation-enhancing properties [18]. In addition, it is easily spreadable and removable and has a pleasing appearance [19]. Nanoemulgels are an attractive technique to overcome these restrictions by integrating oil droplets of nano-size into a hydrogel matrix. This procedure promotes the stability of the formulations, improves the dispersion of lipophilic oils, offers sufficient rheological qualities for topical application, and permits the homogeneous distribution of bioactive elements throughout the formulation. Therefore, nanoemulgels have been widely studied as delivery vehicles for plant-derived oils in medicinal and cosmetic fields [16].

*Linum usitatissimum* (*L. usitatissimum*) (flaxseed) oil belongs to the family *Linaceae*, a natural rich source of α-linolenic acid, linoleic acid, lignans, tocopherols, and phenolic compounds [20] with antioxidant, anti-inflammatory, antimicrobial, and skin-protective activities [21], making it an attractive candidate for topical cosmetic and dermatological applications. However, direct inclusion of flax seed oil into standard topical formulations is limited due to its poor aqueous dispersibility, susceptibility to oxidative degradation, limited skin penetration, and physicochemical instability that may affect its therapeutic and cosmetic performance [22]. Nanoemulgel systems incorporate the benefits of nanoemulsions and gel-based formulations, giving increased solubilization of lipophilic oils, enhanced skin permeability, greater physical stability, controlled release, and better patient acceptability [23,24].

Recent advances in nanotechnology have enhanced the pharmacological and cosmeceutical applications of flaxseed (*Linum usitatissimum*) oil via improving its water dispersibility, physicochemical stability and topical administration. Several researchers have successfully engineered nanoemulsions of flaxseed oil using high-energy homogenisation or ultrasonication. The resultant nanoemulsions of flaxseed oil showed nanosized droplets, relatively small polydispersity indexes and negative zeta potentials, implying good physical stability and increased bioavailability of the hydrophobic drug. A nanoemulsion of flaxseed oil for the management of ulcerative colitis was reported to have nanosized droplets ranging from approximately 156–509 nm, negative zeta potentials and good stability properties, thus showing the potential of a stable nanoemulsion of flaxseed oil for oral pharmaceutical application. However, this study was aimed at oral pharmacological application rather than topical or cosmeceutical application, and the study did not evaluate any dermatological attributes [25].

On similar lines, nanoemulsions of linseed oil have been investigated for their potential as topical pharmaceutical nanocarriers for the management of atopic dermatitis. The linseed oil nanoemulsions were prepared by ultrasonic emulsification and were evaluated for their droplet size, thermodynamic stability and in vitro permeation properties. Their biological evaluation was focused on their potential for topical pharmaceutical application for the management of atopic dermatitis. The nanoemulsions of linseed oil were not aimed at cosmeceutical application with multifunctional attributes such as antioxidant, anti-ageing, antibacterial and sun protection functions [26].

A phytocosmeceutical microemulgel containing flaxseed extract was recently developed by incorporating an oil-in-water microemulsion into a Carbopol gel. The microemulgel developed was found to possess a droplet size of approximately 99 nm, with relatively good polydispersity and zeta potential, it showed antioxidant, tyrosinase inhibition, SPF evaluation, antioxidant, tyrosinase inhibition, SPF evaluation and good cosmetic properties when tested on healthy human volunteers. However, this formulation contained flaxseed extract rather than flaxseed oil, and the study did not evaluate any antibacterial properties or enzyme inhibitory potentials relevant to cosmeceutical application, such as tyrosinase inhibition for skin whitening or elastase inhibition for anti-ageing or α-amylase inhibition for management of obesity [27].

Other researchers have worked on nanoemulgels of seed oils, including flaxseed oil, for the management of dermatological conditions. The studies focused on evaluating physicochemical properties of the nanoemulgels as well as their antioxidant potentials and tyrosinase inhibitory effects. The nanoemulgels showed good stability in terms of physicochemical properties. However, since these studies were focused on a combination of seed oils rather than flaxseed oil alone, it was not possible to determine the specific effects of flaxseed oil. Additionally, there was no optimization of surfactants used in the nanoemulsions, and no comprehensive multifunctional biological evaluation was carried out [28].

Therefore, it can be concluded that despite the growing interest in flaxseed nanoformulations, there is still limited information concerning them. Most previous studies have either focused on formulation development and physicochemical evaluation or have only evaluated a narrow range of biological activities. To the best of our knowledge, no previous study has systematically optimized the preparation of flaxseed oil nanoemulsion using pseudo-ternary phase diagrams and surfactant optimization, followed by incorporation into a nanoemulgel and comprehensive evaluation of multifunctional biological activities, including antioxidant, α-amylase inhibitory, elastase inhibitory, antimicrobial and Sun Protection Factor (SPF) in a single formulation. Therefore, the present study was designed to prepare an optimized nanoemulgel of flaxseed oil with improved physicochemical properties and to evaluate its multifunctional biological activities relevant to topical and cosmeceutical applications.

## 2. Materials and methods

### 2.1. Materials

Dimethyl sulfoxide (DMSO) was acquired from Riedel De Haen (Germany). Al-Shams Company was the source of Tween 20, Tween 80, and Span 80. Carbomer 940 (Carbopol) was obtained from Sinopharm Chemical Reagent Co., Ltd. (Shanghai, China). Trolox ((s)-(-)-6-hydroxy-2, 5, 7, 8-tetramethylchroman-2-carboxylic acid) was acquired from Sigma-Aldrich Denmark, while 2,2-Diphenyl-1-picrylhydrazyl (DPPH) was obtained from Sigma-Aldrich Germany. Laboratory-grade reagents were employed in all experiments. The *L. usitatissimum* seeds that were collected were stored in the pharmacognosy laboratory at the Faculty of Pharmacy, An-Najah National University, in individual herbarium wooden frames and appropriate glassware. Later, the pharmacognosist at the faculty identified them.

### 2.2. *L. usitatissimum* oil extraction

A total weight of 1000 g *L. usitatissimum* seeds was used. The seeds were prepared for extraction by the addition of 200 mL n-hexane and 400 mL 50% ethanol in tripled-distilled water. The mixture was shaken at 200 rpm for 72 h at room temperature and subsequently filtered using a Buchner funnel and suction flask. The filtrate was placed in a separatory funnel to obtain the aqueous (lower) and organic phases (upper; containing the plant oil).

To remove the organic solvent, the organic phase was treated in a rotary evaporator at 40°C for 1 h. After complete drying, the oil extract was stored at 25°C until use. The solid material that remained following the first extraction was re-extracted by the addition of 50 mL hexane and 125 mL 50% ethanol in triple distilled water using the same steps as described above.


$$\begin{array}{c} \text{ Yield }(\%)=\frac{M}{Bm}\;\times \text{1}\text{0}\text{0} \end{array}$$

Where *M* is the mass of the extracted oil (g) and *Bm* is the initial plant biomass (g).

### 2.3. Antioxidant testing of *L. usitatissimum* seed oil

The antioxidant activity of the *L. usitatissimum* seed oil was evaluated using the free radical-scavenging assay. DPPH (1,1-diphenyl-2-picrylhydrazyl) was used to measure the scavenging activity of the oil. The procedure was as follows:

#### 2.3.1. Serial dilutions of *L. usitatissimum* seed oil.

Stock solutions of the *L. usitatissimum* seed oil and trolox (as a standard reference compound) were prepared at a concentration of 1 mg/10 mL, from which serial dilutions were carried out (2, 5, 10, 20, and 50 μg/mL).

#### 2.3.2. Spectrophotometric analysis.

The DPPH solution, as a free radical source, was freshly prepared at a concentration of 0.002% w/v and mixed with methanol and each of the serial solutions at a ratio of 1:1:1. The blank solution was composed of methanol and DPPH at a ratio of 2:1. All samples were incubated in the dark for 30 min at room temperature. The absorption was recorded at a λ_max_ of 715 nm.

#### 2.3.3. Percentage inhibition of DPPH by *L. usitatissimum* seed oil.

The following equation was used to calculate the percentage DPPH inhibition by the trolox standard and the *L. usitatissimum* seed oil:


$$\begin{array}{c} \text{DPPH inhibition }(\%)=(\text{B}-\text{T})/\text{B}\times \text{100} \end{array}$$

Where B is the absorbance of the blank and T is the absorbance of the tested sample.

The antioxidant IC_50_, which represents the concentration of the sample that inhibits 50% of the radicals, was calculated for both trolox and the *L. usitatissimum* seed oil using BioDataFit edition 1.02 [29].

### 2.4. Assessment of elastase inhibition of *L. usitatissimum* seed oil

The spectrophotometric method was used for the assessment of porcine pancreatic elastase (PPE) activity using SANA as the substrate. The procedure was performed as follows:

#### 2.4.1. Preparation of serial dilutions of *L. usitatissimum* seed oil.

Stock solutions of oleanolic acid, as a reference standard, and the *L. usitatissimum* seed oil (each dissolved in 10% DMSO) were prepared at a concentration of 1000 µg/mL, from which serial dilutions were carried out (10, 50, 80, 100, 200, and 500 µg/mL). A solution of elastase was prepared at a concentration of 10 µg/mL dissolved in 10% DMSO. In addition, 5 mM SANA and 2 mM Tris-HCl buffer, pH 8.0, were also prepared.

#### 2.4.2. Spectrophotometric analysis.

Volumes of 5 mL Tris-HCl buffer, 1 mL each serial dilution, and 0.5 mL elastase were mixed and pre-incubated for 10 min at 25°C. Subsequently, 0.5 mL SANA substrate was added to each tube and incubated for a further 15 min at 25°C. The blank solution consisted of all the previous components except the enzyme inhibitor. The absorbance was measured at a λ_max_ of 410 nm.

#### 2.4.3. Percentage of elastase inhibition.

The anti-aging effect of the *L. usitatissimum* seed oil nanoemulgel was demonstrated by measuring the ability of the oil to inhibit elastase activity in comparison with the standard using the following equation [30]:


$$\begin{array}{c} \text{Inhibition }(\%)=\left(\text{1}-B/A\right)\times \text{100} \end{array}$$

Where A is the enzyme activity without the presence of oil (blank solution) and B is the enzyme activity in the presence of the inhibitor.

### 2.5. Measurement of the SPF of *L. usitatissimum* seed oil

A sample of *L. usitatissimum* seed oil was prepared for the measurement of the SPF value. A mass of 1 g oil extract was added to 100 mL ethanol in a volumetric flask and subjected to ultrasonication. To calculate the SPF value, the absorption was measured spectrophotometrically at wavelengths of 290 − 320 nm using the Mansour equation:


$$\begin{array}{c} \text{SPF}=\text{CF}\times \Sigma \text{ EE }(\lambda )\times \text{I }(\lambda )\times \text{Abs }(\lambda ) \end{array}$$

Where CF is the correction factor, which equals 10; EE (λ) is the erythmogenic effect of radiation at wavelength λ; Abs (λ) is the spectrophotometric absorbance value at wavelength λ; and I (λ) is the solar intensity spectrum at wavelength λ.

### 2.6. Preparation of the nanoemulgel

The nanoemulgel was produced by the incorporation of the nanoemulsion to the hydrogel in sequential steps:

#### 2.6.1. Preparation of *L. usitatissimum* seed oil nanoemulsion.

*L. usitatissimum* seed oil nanoemulsions were prepared using three different elements at different concentrations using the self-emulsifying technique. These elements were the *L. usitatissimum* seed oil, Tween® as the surfactant, and Span® as the co-surfactant. The self-emulsifying system is a mixture of natural or synthetic oils, solid or liquid surfactants, and co-surfactants with isotropic behavior [31], so called due to the spontaneous emulsification when mixing with the aqueous phase upon gentle agitation [32]. The mechanism of this system can be explained by the fact that the energy required to increase the surface area of dispersion is lower than the entropy change, favoring dispersion. In addition, the energy required to add a new surface between the two immiscible phases, oil and water, is known as the emulsion free energy, which tends to decrease over time as the emulsion phases separate [33].

To optimize the nanoemulsion formulation, a ternary phase diagram was constructed using the three elements mentioned above. Each formulation was measured using a sensitive balance and subsequently vortexed (CLASSIC Advanced Vortex Mixer) for 2 min. Emulsification of the oil with water under gentle agitation was then performed. Finally, the droplet size and the polydispersity index (PDI) were measured, and the optimum formulation was selected based on these characteristics and the formulation with the highest oil concentration for subsequent nanoemulgel preparation.

**2.6.1.1 Analysis of the nanoemulsion characteristics:** A NanoBrook Omni 280173 (Brookhaven, New York) was used to determine the polydispersity index (PDI) and droplet size of the nanoemulsion. The mean and standard deviation were measured in triplicate. The formulation that obtained the smallest droplet size, with the lowest PDI and the highest amount of oil, was selected.

#### 2.6.2. Hydrogel formulation.

The hydrogel was created by the addition of Carbopol® 940 to water and continuous mixing using a homogeniser to prepare a uniform dispersion. Subsequently, under constant mixing, 2M NaOH was added to the hydrogel to adjust the pH to 6. The mixture underwent continuous mixing and was left for 24 h to completely gel.

#### 2.6.3. Nanoemulgel formulation.

The nanoemulgel formulation was prepared by the addition of different concentrations (0.4, 0.6, and 0.8%) of the hydrogel matrix to the optimum nanoemulsion at 100 rpm for 10 min. The polydispersity index, droplet size, and zeta potential were subsequently measured.

**2.6.3.1 Physical characterization of the nanoemulgel:** The droplet size, polydispersity index, and spreadability, in addition to the visual appearance of the nanoemulgel formulation were inspected.

**2.6.3.2 Analysis of the zeta potential of the nanoemulgel:** The zeta potential is defined as the potential that forms between the oil droplet and the liquid phase. Stable formulations have a positive or negative potential greater than 30 mV. A NanoBrook Omni 280173 (Brookhaven, New York) was used to measure the zeta potential of the nanoemulgel formulation.

**2.6.3.3 Rheological measurement of the nanoemulgel:** The rheological performance of the emulgel formulations prepared with different concentrations of Carbopol® 940 (0.4, 0.6, and 0.8%) as a gelling agent was evaluated at ambient temperature using a rotational viscometer (Brookfield DV1, USA). All measurements were performed in triplicate. The viscosity was determined within the shear-rate range (0 − 100 rpm).

### 2.7. Antimicrobial test

#### 2.7.1. Microorganisms.

The organisms used for the bacterial test were *Escherichia coli* (*E. coli*) (ATCC 25922), *Staphylococcus aureus* (*S. aureus*) (ATCC 25923), Methicillin-Resistant *Staphylococcus Aureus* (MRSA), *Shigella sonnei* (*S. sonnei*) (ATCC 25931), and *Enterococcus faecium* (*E. faecium*) (ATCC 700221), and *Candida albicans* (*C. albicans*) (ATCC 90028) was used for the fungal test.

#### 2.7.2. Culture media.

The bacterial culture medium was Muller Hinton agar, which is nonselective and contains 2 g beef extract, 17.5 g acid hydrolysate of casein, 1.5 g starch, and 17 g agar per liter of purified water. The media was made by mixing the components together and heating with agitation until boiling. Subsequently, the media was autoclaved for 20 min at 121°C. After cooling, the agar was poured into sterile petri dishes on a flat surface to obtain depth uniformity and stored at 4°C. The fungal culture media was Sabouraud dextrose agar, which contains 40 g of dextrose, 10 g of peptone, and 20 g of agar per liter of distilled water. The media was prepared as described above.

McFarland turbidity standards were used to standardise the inoculums. McFarland 0.5 standard was used for turbidity comparison, which offers a turbidity similar to that of a bacterial suspension containing 1.5 x 10^8^ CFU/ml.

#### 2.7.3. Agar well diffusion test.

Antimicrobial activity of formulations was assessed using the agar well diffusion method. Sterile Mueller–Hinton agar plates (or Sabouraud dextrose agar for *Candida albicans*) were inoculated with microbiological suspensions calibrated to 0.5 McFarland standard (about 1.5 × 10^8^ CFU/mL for bacteria). Wells of 6 mm diameter were aseptically created in the agar, and 100 µL of each formulation was carefully inserted in each well. The plates were incubated at 37°C for 24 h with bacterial strains and at 30°C for 48 h with *Candida albicans*. Fluconazole (50 µg/mL) was used as the positive antifungal control, while the blank formulation was used as the negative control. The antimicrobial activity was evaluated by measuring the diameter of inhibitory zones (mm). Each experiment was done in triplicate (n = 3), and the entire experiment was repeated independently three times. The results are expressed as mean ± standard deviation (SD).

### 2.8. Statistical analysis

All experiments were carried out in triplicates, and findings are represented as mean ± standard deviation (SD). Statistical analysis was performed using SPSS. One-way analysis of variance (ANOVA) with Tukey’s multiple comparison post hoc test was used to compare between multiple treatment groups. Comparisons between two groups, where applicable, were performed using an unpaired Student’s t-test. Statistical significance was defined as p value of less than 0.05.

## 3. Results

### 3.1. Percentage yield of *L. usitatissimum* seed oil

The total weight of the *L. usitatissimum* seed used for extraction was 1000 g, from which 15 g oil was obtained, equal to 1.5% yield.

### 3.2. Antioxidant activity of *L. usitatissimum* seed oil

The antioxidant activity of *L. usitatissimum* seed oil is related to the scavenging activity of free radicals. The results are shown in Table 1 and explained in Fig 1.

**Table 1:** The comparison between Trolox standard and *L. usitatissimum* seeds oil according to the % of Inhibition.

Concentration (µg/ml)	%age Inhibition by Trolox	%age Inhibition by *L. usitatissimum* oil
100	97.12 ± 2.13	65.18 ± 2.36
50	92.30 ± 2.71	63.18 ± 1.66
20	87.25 ± 2.13	58.53 ± 2.32
10	83.22 ± 2.25	57.53 ± 1.89
5	75.80 ± 3.02	55.15 ± 2.86
2	58.12 ± 2.88	44.47 ± 2.57
IC_50_	2.21 ± 0.23 µg/ml	3.15 ± 0.46 µg/ml

**Fig 1:**
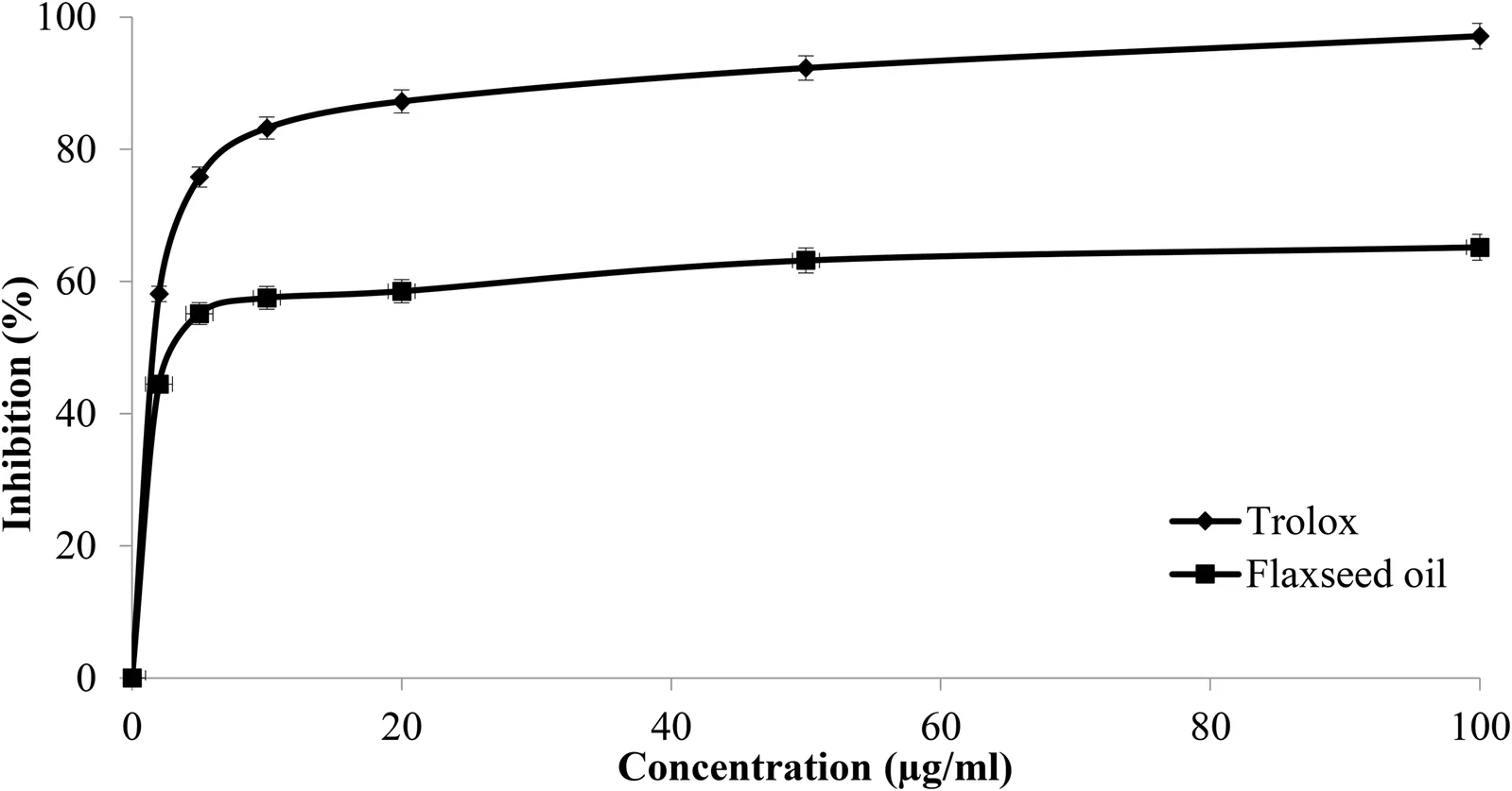
Inhibition activity of *L. usitatissimum* seed oil compared to Trolox standard.

### 3.3. Elastase inhibition activity of *L. usitatissimum* seed oil

As shown in Fig 2, the elastase inhibition activity of the *L. usitatissimum* seed oil revealed an IC_50_ of 17.3 ± 0.5 µg/ml. The IC_50_ of oleanolic acid (as a reference standard) was 10.2 ± 0.3 µg/ml.

**Fig 2:**
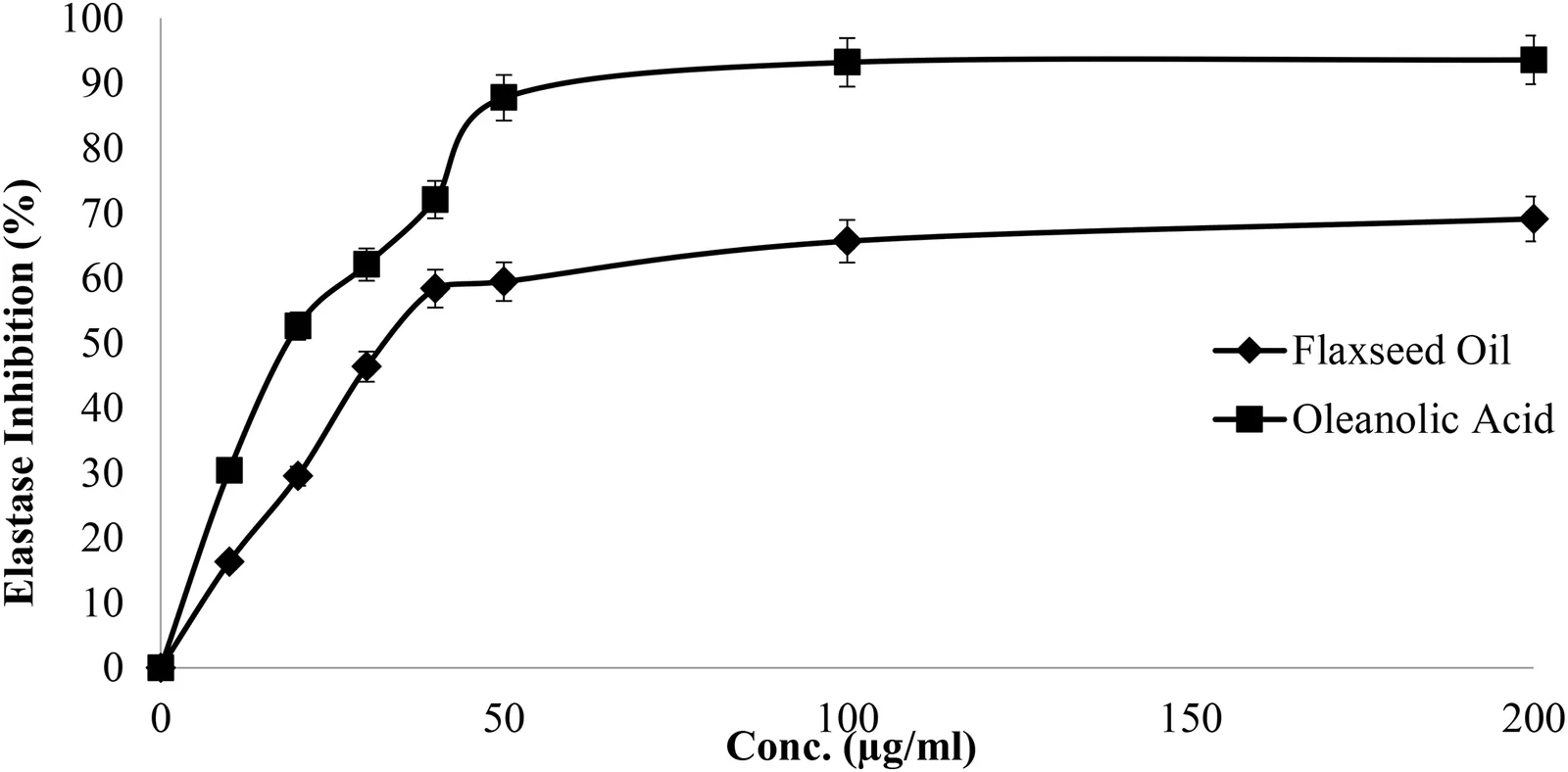
Elastase enzyme inhibition profile for *L. usitatissimum* seeds oil.

### 3.4. SPF (Sun Protection Factor) of *L. usitatissimum* seed oil

Using the SPF equation, the SPF value of *L. usitatissimum* seed oil was found to be 5.87 (Table 2).

**Table 2:** Normalized product function used in the SPF calculation.

Wavelength (λ nm)	EE x 1 (Normalized)	Absorption
290	0.0150	0.667
295	0.0817	0.772
300	0.2874	0.688
305	0.3278	0.561
310	0.1864	0.482
315	0.0839	0.429
320	0.0180	0.372

### 3.5. Characterization and selection of the optimum *L. usitatissimum* nanoemulsion

In the initial process, several nanoemulsion formulations with various concentrations of Tween® 80, Tween® 20, Span® 80, and *L. usitatissimum* seed oil were created and tested. The formulations were evaluated for their capacity to generate homogenous nanoemulsions inside the nanoemulsion region found from the pseudo-ternary phase diagram. Prepared systems were characterised for mean droplet size, polydispersity index (PDI), zeta potential, visual appearance and physical stability. Of the formulations investigated, the formulation with 35% Tween® 80, 5% Span® 80 and 60% *L. usitatissimum* seed oil exhibited the most favorable physicochemical characterization, including the smallest droplet size, narrow size distribution and highest oil concentration in the formulation. Thus, this formulation was chosen as the optimized nanoemulsion for further integration into the Carbopol® gel matrix. Fig 3 represents the phase diagrams of *L. usitatissimum* seed oil nanoemulsions constructed using Tween® 80/Span® 80 and Tween® 20/Span® 80, respectively.

**Fig 3:**
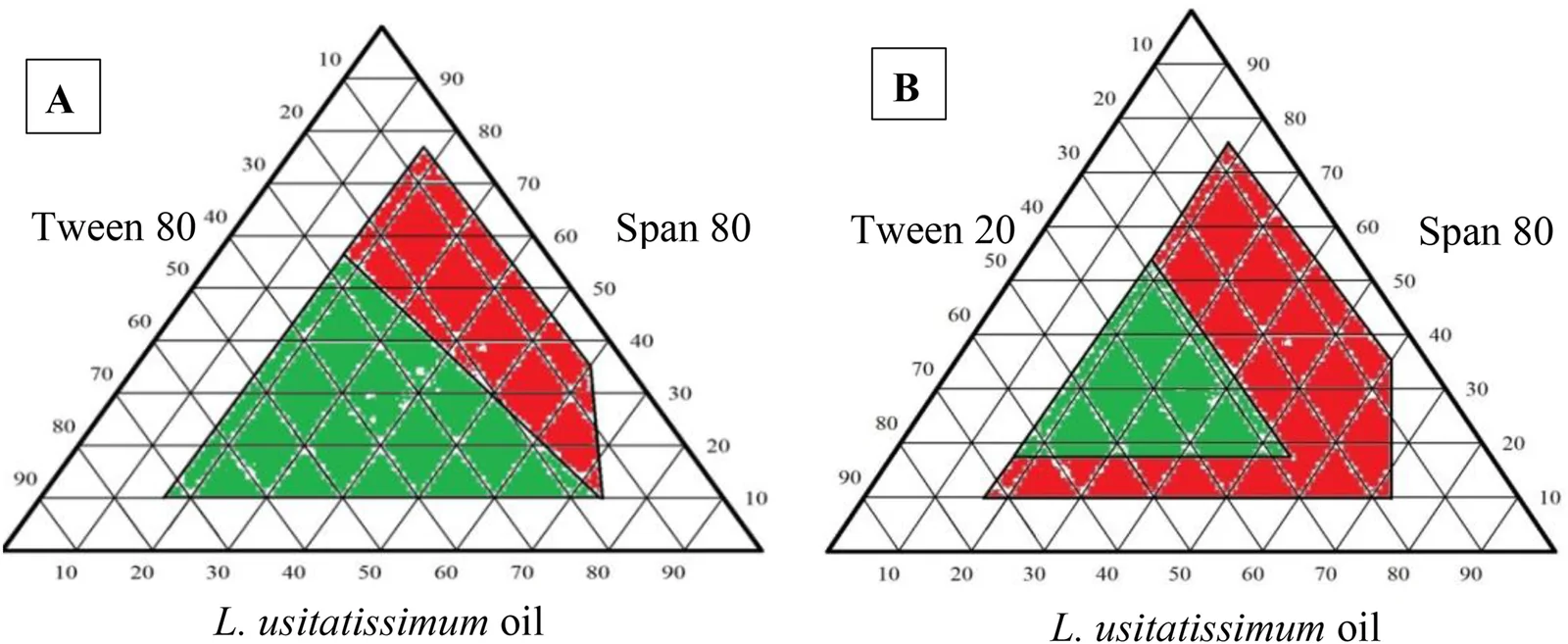
Ternary phase diagrams of *L. usitatissimum* seeds oil nanoemulsion construct with A: Tween 80 and Span 80 and B Tween 20 and Span 80.

Both surfactants showed different behaviors. Tween® 80 was able to produce an emulsion with a droplet size less than 200 nm and a large nanoemulsion region as compared with Tween® 20. The optimum formulation was prepared from 35% Tween® 80, 5% Span® 80, and 60% *L. usitatissimum* seed oil, showing a droplet size of 152 ± 4.32 nm and a polydispersity index of 0.211 ± 0.02.

### 3.6. Nanoemulgel of *L. usitatissimum* seed oil formulations

Nanoemulgel formulations containing *L. usitatissimum* seed oil were prepared using different concentrations of Carbopol® 940 (0.4, 0.6, and 0.8%) as a gelling agent, which provides swelling properties to the formulation [34]. A self-emulsifying technique was used to produce the optimum nanoemulsion formulation, and Carbopol® hydrogel was subsequently added under continuous stirring to form the nanoemulgel. The droplet size, polydispersity index, zeta potential, and rheological behavior of the nanoemulgel formulations were measured.

### 3.7. Effect of Carbopol® on the droplet size and polydispersity index of the nanoemulgel

The droplet size (Fig 4) and polydispersity index (Fig 5) of the nanoemulgel formulations containing different Carbopol® concentrations (0.4%, 0.6%, and 0.8%) were compared with the initial nanoemulsion. Statistical analysis did not show significant differences (p > 0.05) in droplet size and polydispersity index of the nanoemulgel formulations prepared with different Carbopol® concentrations, suggesting that incorporation of the gel matrix did not negatively affect these characteristics of the optimized nanoemulsion.

**Fig 4:**
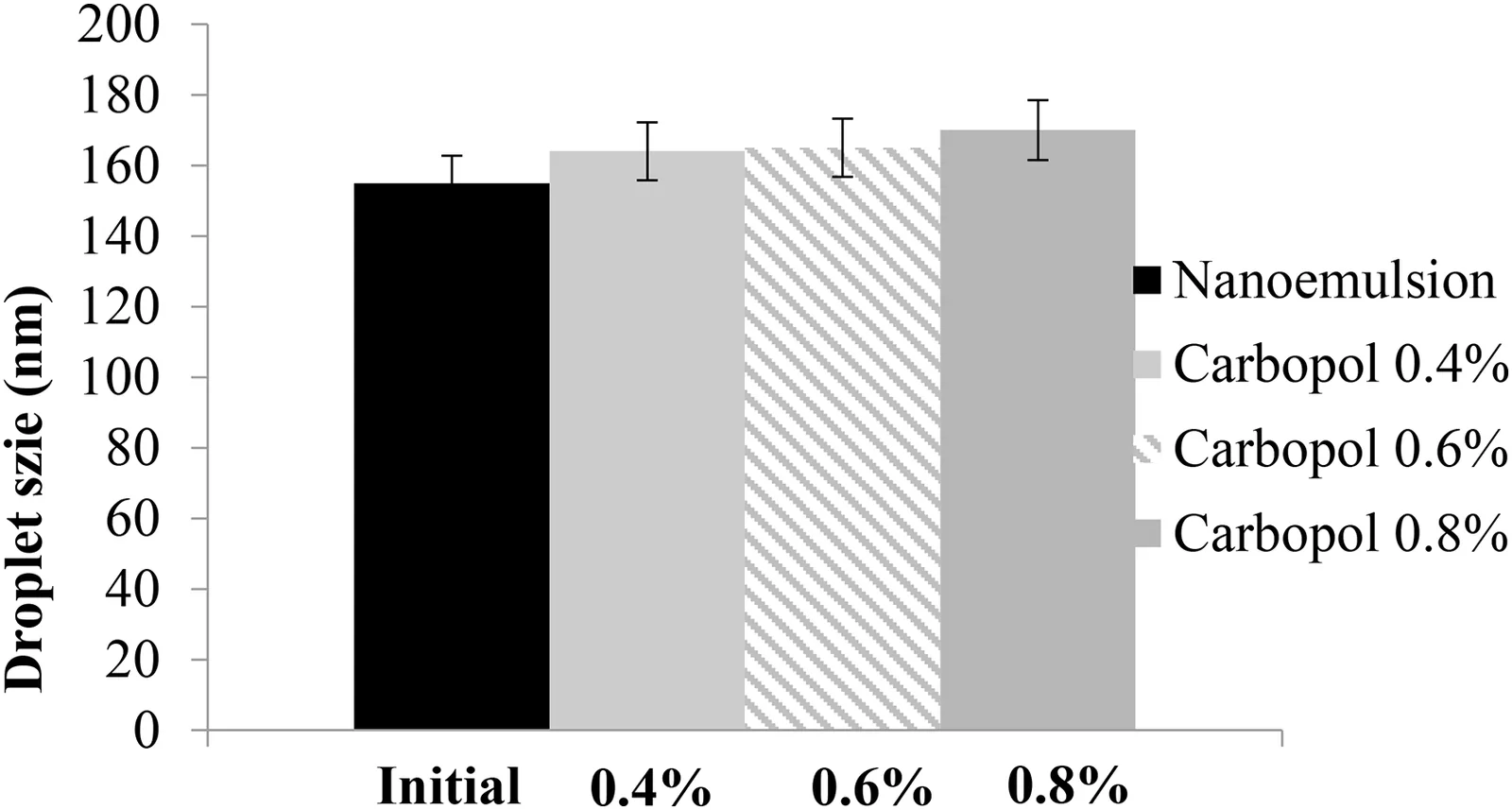
Droplets size of initial *L. usitatissimum* seed oil nanoemulsion and nanoemulgel formulations containing different concentrations of Carbopol (0.4, 0.6, 0.8%).

**Fig 5:**
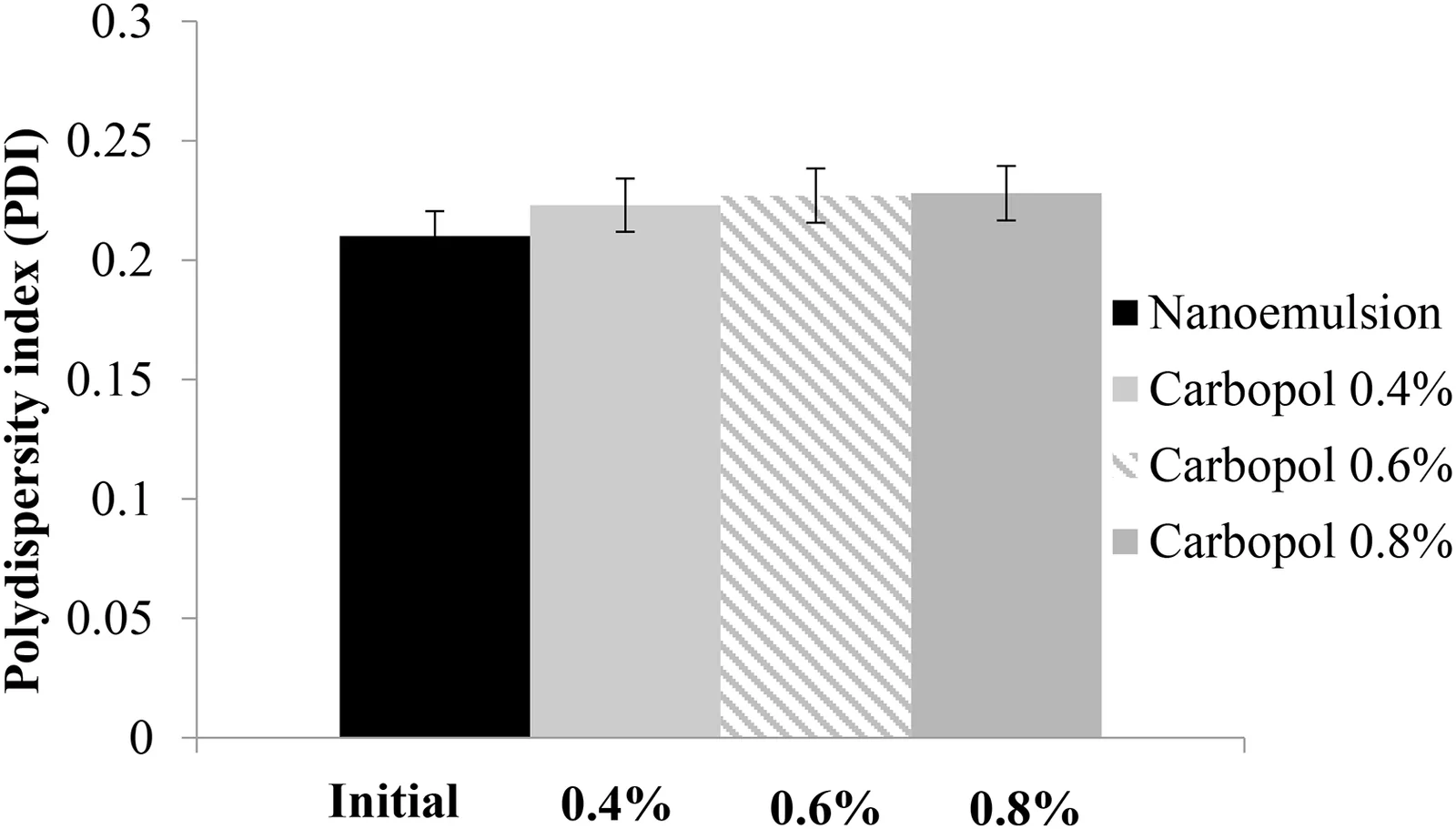
Polydispersity index measurement of initial *L. usitatissimum* seed oil nanoemulsion and nanoemulgel formulations containing different concentrations of Carbopol (0.4, 0.6, and 0.8%).

### 3.8. Zeta potential of the nanoemulgel

The zeta potential values of the nanoemulgel formulations were found to be −35.4 ± 1.1 mV, −35.8 ± 0.9 mV and −36.2 ± 1.3 mV, for nanoemulgel formulations containing different concentrations of Carbopol 0.4, 0.6, and 0.8%, respectively. The zeta potential of all the formulations was more negative than −30 mV, indicating a substantial electrostatic repulsion between the droplets with a satisfactory colloidal stability. Statistical analysis using one-way ANOVA demonstrated that there were no significant differences in zeta potential among the three nanoemulgel formulations containing 0.4%, 0.6%, and 0.8% Carbopol® (p > 0.05), indicating that variation in Carbopol® concentration did not significantly influence the electrostatic stability of the nanoemulgels.

### 3.9. Rheological behavior of the nanoemulgel

Pharmaceutical products, especially semisolids forms, are typically evaluated according to the rheological properties (flow properties), which are an important indicator of the quality and efficacy of the formulation. Rheological analysis of the nanoemulgel showed an increase in the shear rate and a decrease in the viscosity, indicating that the behavior of the nanoemulgel is pseudoplastic (Fig 6).

**Fig 6:**
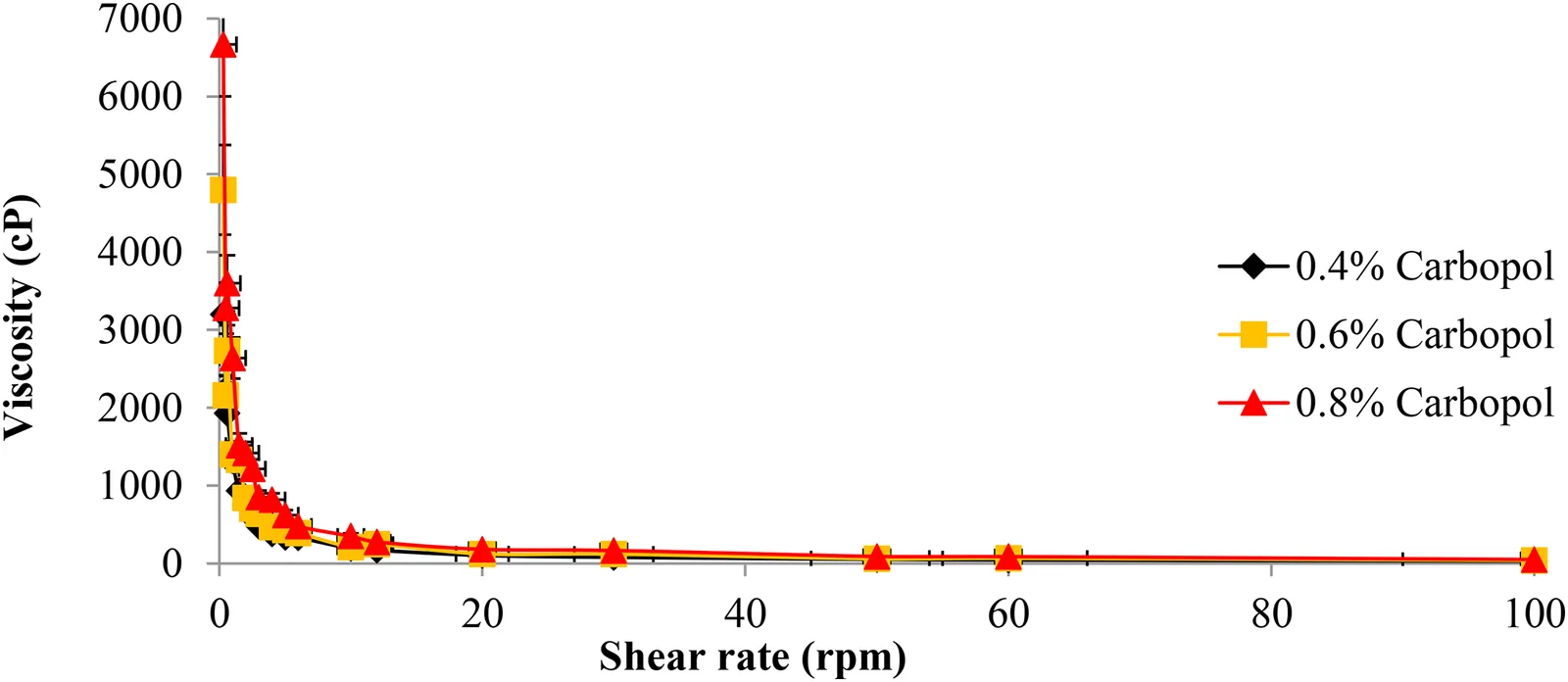
Rheological behavior of different *L. usitatissimum* seed oil nanoemulgel formulations containing different concentrations of Carbopol (0.4, 0.6, and 0.8%).

### 3.10. Spreadability properties of the nanoemulgel

Three different *L. usitatissimum* seed oil nanoemulgels containing different concentrations of Carbopol® (0.4, 0.6, and 0.8%) were evaluated with respect to the spreadability on the skin and the ease of removal from the jar. It was found that the emulgel containing 0.4% Carbopol® was removed from the jar most easily. The spreadability was similar for all the formulations; however, the emulgel containing 0.4% Carbopol® was chosen, since it has the lowest Carbopol® content.

### 3.11. Antimicrobial activity

The antibacterial activity of crude *L. usitatissimum* oil and the optimized nanoemulgel formulation against the tested microorganisms was assessed using the agar well diffusion technique. Table 3. Zone of inhibition diameter (mean ± SD). The results of one-way ANOVA followed by Tukey’s post hoc test indicated that the inhibitory zones of the nanoemulgel formulation against *S. aureus* and MRSA were significantly bigger than the crude oil (p < 0.05). The nanoemulgel inhibited *S. aureus* in a similar manner to the positive control (Ampicillin), and there was no significant statistical difference between these two treatments (p > 0.05).

**Table 3:** Antimicrobial activities (Inhibition zone (cm ± SD)) of *L. usitatissimum* seed oil and its nanoemulgel against Ampicillin and Fluconazole.

Microorganism	*L. usitatissimum* oil	*L. usitatissimum* oil nanoemulgel	Ampicillin	Fluconazole
**MRSA**	3.1 ± 0.14^a^	4.2 ± 0.14^b^	4.5 ± 0.03^b^	--
***S. aureus***	2.5 ± 0.10^a^	3.7 ± 0.11^b^	3.7 ± 0.03^b^	--
***E. faecium***	No effect	No effect	1.8 ± 0.04	--
***E. coli***	No effect	No effect	3.3 ± 0.08	--
***S. sonnei***	No effect	No effect	4.0 ± 0.07	--
***C. albicans***	1.4 ± 0.13^a^	1.9 ± 0.09^b^	--	1.2 ± 0.01^c^

Values are expressed as mean ±SD (n = 3). Different superscript letters within each row indicate statistically significant differences according to one-way ANOVA followed by Tukey’s multiple comparison test (p < 0.05).

The crude oil and the nanoemulgel did not show antibacterial activity against *E. coli*, *E. faecium*, and *S. sonnei*. *C. albicans* exhibited mild suppression, and statistical analysis revealed that nanoemulgel exhibited considerably more inhibition than crude oil (p < 0.05) but was less active than fluconazole (p < 0.05).

## 4. Discussion

In the present study, *L. usitatissimum* seed oil nanoemulgel was prepared with a view to investigating its cosmeceutical and pharmaceutical applications. During the first evaluation stage, the SPF and antioxidant activity of the oil were investigated. Subsequently, the *L. usitatissimum* seed oil nanoemulgel was prepared using the self-nanoemulsifying technique, followed by the incorporation of Carbopol® 940 hydrogel. The physical and antimicrobial properties of the nanoemulgel were investigated.

Regarding the physical characteristics of the prepared nanoemulgel, it had the smallest droplet size and polydispersity index. The zeta potential was in the stable range acceptable for nanoemulsion, with a very short dispersion time of the oil droplets (6 seconds). In addition, it had a homogeneous visual appearance. Moreover, the color of the nanoemulgel was white, and the viscosity was suitable to give good spreadability and a smooth, creamy behavior.

The antioxidant and anti-ageing results, in addition to the SPF value, of the nanoemulgel supported its utilization in cosmeceutical applications. The prepared formulation was shown to have a high free radical scavenging activity, as demonstrated by its IC_50_ in comparison with the trolox standard (3.15 ± 0.46 and 2.21 ± 0.23 µg/mL, respectively). There is accumulating evidence suggesting an antioxidant activity of *L. usitatissimum* seeds and their extracts. In most studies, the antioxidant activity is significantly correlated with the phenolic content [35]. In 2010, Zanwar et al., demonstrated the concentration-dependent antioxidant activity of *L. usitatissimum* seeds using DPPH scavenging activity [36]. In 2017, Yilmaz et al., also determined the antioxidant activity of this plant extract using different methods [37]. The antioxidant activity observed in the present study is consistent with previous investigations demonstrating that *L. usitatissimum* seed oil possesses remarkable free radical scavenging properties owing to its high content of lignans, phenolic acids, tocopherols, and α-linolenic acid. The IC₅₀ value obtained in our study (3.15 ± 0.46 μg/mL) is comparable to those reported for flaxseed extracts and oils using DPPH and ABTS assays, indicating that the extraction procedure successfully preserved the bioactive antioxidant constituents. Minor variations among published studies are expected because antioxidant activity is influenced by cultivar, geographical origin, extraction solvent, oil processing, and analytical methodology. Compared with other plant oils used in topical nanoformulations, the antioxidant capacity of *L. usitatissimum* oil is among the highest reported, supporting its suitability for cosmeceutical applications aimed at reducing oxidative skin damage.

The antioxidant activity observed for the crude flaxseed oil was found to be in line with recently reported results demonstrating the bioactive value of *L. usitatissimum* lipid components. Incorporating this oil into nanostructures holds promise for protecting its high content of polyunsaturated fatty acids from oxidative degradation, thereby supporting the rationale for developing a nanoemulgel system. Indeed, the studied oil is traditionally reported to contain an impressive amount of α-linolenic acid, tocopherols, and lignan derivatives, while its oxidative stability is considerably limited due to the high content of polyunsaturated fatty acids. The recent findings have revealed that the production of flaxseed oil nanoemulsions containing biologically active phenolic compounds resulted in an improvement of their oxidative stability likely due to the increased protection of unsaturated compounds and optimized distribution of antioxidant components at the oil-water interface [38]. Similarly, the antioxidant activity of the present nanoemulgel may be rationalized by the combined contribution of the antioxidant compounds contained in the flaxseed oil and the nanostructure of the formulation that facilitates their dispersion in the aqueous medium and, consequently, their availability for interaction with the radical scavenging molecules. It was recently shown that a flaxseed-based phytocosmeceutical microemulgel exhibited an important antioxidant activity and simultaneously enhanced cosmetic properties, therefore qualifying as a multifunctional cosmetic formulation [27]. Compared with previously reported conventional flaxseed oil systems, the improved antioxidant activity demonstrated by the formulated system proves the efficacy of nanoemulsion/nanoemulgel system in extending the application of lipidic botanicals for cosmeceutical purposes.

Elastase is a protease enzyme belonging to the chemotrypsin family of proteins. It is responsible for the degradation of elastin and other proteins typically found in the extracellular matrix (ECM) [39]. Increased levels of elastase are normally associated with the accumulation of elastic fibers in the skin’s dermis, thus reducing its elasticity [40]. As a result, the enzyme is considered an important factor in skin aging (development of wrinkles); therefore, any product with an inhibitory activity against this enzyme is concluded to have good anti-aging properties in cosmetics [41]. The elastase inhibitory effect of *L. usitatissimum* oil further supports its potential use as an anti-aging substance. The IC_50_ observed in the present investigation is comparable with some plant extracts previously studied for cosmetic applications. Oleanolic acid remains a more effective reference inhibitor, however, *L. usitatissimum* seed oil is surprising as an inhibitor considering it is a crude natural oil and not a refined active ingredient. Elastase inhibition has been previously attributed to flaxseed lignans along with phenolic compounds that can reduce elastase-mediated breakdown of extracellular matrix proteins. The present investigation showed rather substantial inhibitory activity that supports the use of flaxseed oil as a multifunctional agent providing both antioxidant protection and preservation of skin elasticity.

The elastase inhibitory potential demonstrated by the crude flaxseed oil (IC_50_: 17.3 ± 0.5 µg/mL) provides a strong rationale for incorporating this oil into anti-aging cosmeceutical formulations. While the elastase assay in this study evaluated the raw seed oil, the encapsulation of such bioactive botanical oils into nanoemulgel systems is expected to enhance their dermal delivery and bioavailability relative to the free oil. The present compound comprises unsaturated fatty acids, tocopherols, and phenolic acids, which have been implicated in the attenuation of oxidative stress-mediated extracellular matrix remodeling. In addition, the nano-delivery system might improve the bioavailability and dermal penetration of the hydrophobic cargo compared to its free-form counterpart [27].

In the present study, the IC_50_ of the *L. usitatissimum* seed oil nanoemulgel against elastase was found to be 17.3 ± 0.5 µg/mL, and that of the oleanolic acid standard was 10.2 ± 0.3 µg/mL. The results show that the nanoemulgel had more than half the inhibition activity of the standard, which may be related to the high phenolic and flavonoid contents of the plant extract [42]. Sultana et al., conducted a study on Cornus kousa stems and its isolated compounds, maslinic acid, arjunolic acid, and two ellagic acid derivatives, which showed significant inhibition activity against elastase, with IC_50_ values of 10.81 µg/mL, 21.21 µg/mL, and 44.63 µg/mL, respectively [43]. Another study found that a crude grape pomace extract had a dose-dependent enzyme inhibition activity of 14.7 μg/mL [44]. The results of the present study indicate a potential anti-aging activity of the *L. usitatissimum* seed oil by the inhibition of elastase with an IC_50_ of 17.3 ± 0.5 µg/mL.

The SPF value of the nanoemulgel was an indicator of its ability to absorb ultraviolet radiation to protect the skin from the hazard of exposure. As observed, the SPF value of the nonvolatile oils has been found to be between 2 and 8 [45]. Here, the *L. usitatissimum* seed oil SPF was shown to be approximately 5.87; thus, we verified the sun protection activity of the prepared formulation. The pH value of the nanoemulgel was measured to be roughly 6. This value was found to be acceptable for application to the skin with no signs of irritability, since the pH of the skin is approximately 5.6 [46]. The SPF value of 5.87 reported in the present study is in agreement with prior publications identifying natural vegetable oils as offering low to moderate intrinsic photoprotection. This level of protection is not enough to use as a sunscreen on its own but is comparable with SPF values recorded for other cosmetic oils, including olive, sesame, coconut, and flaxseed oils. Thus, oil of *L. usitatissimum* may be a beneficial auxiliary ingredient in multifunctional topical preparations offering antioxidant protection and acting as a supplemental UV protectant.

The observed SPF value of the crude flaxseed oil (5.87) was found to be comparable to previously reported data on natural vegetable oils offering intrinsic low-to-moderate photoprotection. These findings indicate that *L. usitatissimum* seed oil can serve as a beneficial functional oil phase in the formulated nanoemulgel, contributing supplemental UV-protectant and antioxidant qualities to the final topical product. Recent studies have demonstrated that the production of nanoemulsions or microemulgels based on botanical oils and extracts may improve their optical properties and increase their attractiveness for cosmetic topical applications. The microemulgel containing flaxseed extract was found to exhibit an SPF value along with antioxidant and dermatotropic activity, thus, being a promising candidate for the photoprotective cosmetic industry [27]. Nevertheless, the SPF observed in the present study should be considered an in vitro screening value, and further in vivo photoprotection studies are required before clinical claims can be established.

Comparison of the two non-ionic surfactants used in the present study showed that Tween® 80 was able to give a larger nanoemulsion region than Tween® 20. Bouchemal et al., support our results that Tween® 80 gives a smaller droplet size; in this study, both surfactants were used in the preparation of the nanoemulsion, and the droplet size resulting from Tween® 80 was 516 ± 71 nm and from Tween® 20 was 725 ± 198 nm [47]. In a study on Swietenia oil, although Tween® 20 produced good self-emulsification properties due to its good solubility, utilisation alone rather than mixing with other surfactants with a low HLP value gave a small nanoemulsion region. The explanation given was that this surfactant has a large number of hydrophilic groups that result in immiscible oil/surfactant mixtures, therefore producing poor self-emulsification properties with hydrophobic oil [48].

Many studies have focused on the solubility properties of Tween® 80. An example is the poorly soluble anti-inflammatory drug Piroxicam; its solubility was markedly increased by the presence of Tween® 80 as a liquid vehicle [49]. In addition, Tween® 80 solution has been used as a vehicle for the progesterone metabolite, 5α-dihydroprogesterone, after the oily vehicle failed to provide the same efficacy. Tween® 80 solution was able to give a similar efficacy, since it possessed the required solubility properties [50]. A study on NSAID drugs conducted to demonstrate their solubility using a series of Tween® surfactants (80, 60, 40, 20) showed that Tween® 80 gave the highest solubility and Tween® 20 gave the lowest. These results suggested that the solubility of Tween® surfactants was approximately proportional to the surfactant concentration [51]. Moreover, Soon et al., tested the solubility of flurbiprofen in some surfactants, showing that the solubility in Tween® 80 was 11,193.2 ± 3074.9 (μg/mL) and that in Tween® 20 was much lower at 8735.1 ± 1220.6 (μg/mL) [52]. The above-mentioned studies are considered evidence of the ability of Tween® 80 to give a smaller droplet size due to its high solubilizing properties.

The selected formulation in the present study, composed of 35% Tween® 80, had the smallest droplet size of 152.17 ± 4.32 nm and a polydispersity index of 0.211 ± 0.02 nm. These results are in accordance with the nanoemulsion prepared by Mahiran et al., whose optimum formulation had a similar Tween® 80 ratio. In addition, it was found that the significant decrease in droplet size was due to the increased Tween® 80 ratio up to 35%, which was a result of the increase in the surfactant adsorption around the droplet at the oil-water interface and a decrease in the interfacial tension, increasing the favourability of forming the smallest droplet size [53].

As mentioned previously, the difference in the Carbopol® concentrations in the nanoemulgel formulations had no effect on the rheological behavior. Moreover, there were no significant differences in the mean droplet size or polydispersity index of the nanoemulgel formulations in comparison with the optimum nanoemulsion. Similar results were also found by Yilmaz et al., [54]; however, a slight increment in the mean droplet size of the formulations was seen with an increment in the Carbopol® concentration. Similar findings have been stated by different authors, where a higher concentration of the polymer resulted in an increase in the viscosity of the medium, which resulted from a high degree of cross-linking [55,56]. There were no significant variations (p > 0.05) in droplet size, polydispersity index, or zeta potential among the nanoemulgel formulations created with varied amounts of Carbopol®, as revealed by statistical analysis. These data demonstrate that the increase in Carbopol® content in the studied range did not have deleterious effects on the physicochemical stability of the optimized nanoemulsion.

The zeta potential of the nanoemulgel formulations was below −30 mV, which is an indication of stability, since previous reports have shown that emulsions with a positive or negative zeta potential of more than 30 mV are considered stable [48,52]. In addition, the narrow polydispersity index, which is related to the size distribution, validated the high stability of the nanoemulgel formulations. This is supported by Jeong et al., who reported that emulsions with a narrow size distribution less than 0.3 have a higher stability than those with a broader size distribution [57]. This measured parameter showed a similar performance and behavior among all the nanoemulgel formulations; however, the nanoemulgel formulation containing the lowest Carbopol concentration of 0.4% was optimal.

The antimicrobial results obtained in this study are in accordance with the earlier results of the antibacterial activity of bioactive compounds from *L. usitatissimum* seed, particularly cyclolinopeptides and phenolic contents, against Gram-positive microbes. Susceptibility was higher for *Staphylococcus aureus* as compared to Gram-negative bacteria, as in earlier studies, perhaps because of the protective outer layer in Gram-negative organisms, which limits penetration of hydrophobic phytochemicals. Of importance, the addition of *L. usitatissimum* seed oil into the nanoemulgel greatly improved the antibacterial activity as compared to the crude oil. Similar improvements have been observed for nanoemulsion-based delivery systems using essential oils and plant extracts. A decrease in droplet size increases the surface area, which aids the diffusion through the microbial cell walls and boosts the contact with bacterial membranes. Thus, the higher antibacterial activity in this study may be due to the intrinsic antimicrobial activity of the flaxseed oil and the enhanced transport by nanoemulgel formulation [58].

The *L. usitatissimum* seed oil showed antimicrobial activities due to the presence of cyclolinopeptides as one of the antibacterial components in *L. usitatissimum* seed oil [59]. Statistical study revealed that optimized nanoemulgel was significantly more effective in inhibitory zones against *S. aureus*, MRSA and *C. albican*s than crude *L. usitatissimum* seed oil (p < 0.05). The enhanced antibacterial activity is probably due to the decreased droplet size (<200 nm), which increases the accessible surface area for interaction with bacterial cell membranes and enhances penetration into microbial biofilms and cell envelopes. To emphasise this idea, Withania somnifera cream in silver nanoparticle form was evaluated in another study, and an increase in the penetration of the cream was found, increasing the microbial inhibition [60]. Moreover, Lkhagvaja et al., demonstrated that the microbial inhibition of colloidal silver nanoparticles was increased as a result of the preparation method and particle size [61].

The antibacterial activity of the nanoemulgel was due to the close packing and high exposure to the bacteria. As a result, an increased concentration of the *L. usitatissimum* seed oil penetrated the bacteria. To reinforce this idea, Assali et al., found that the antimicrobial effect of ciprofloxacin single-walled carbon nanotubes was due to an increase in the drug residence time as a result of drug aggregation, in turn increasing penetration and inhibition [62].

Recent studies have established the ability of nanoemulsion-based delivery systems to improve the antimicrobial performance of botanical oils. For instance, nanoemulgels produced from natural oils demonstrated enhanced antimicrobial properties compared to their traditional counterparts, underlining the benefits of nanostructuring on the physicochemical characteristics of the excipients and, by extension, their biological functions [63].

Consistently with these findings, the antimicrobial efficacy of the fabricated flaxseed oil nanoemulgel can be explained by the combined contribution of the active compounds derived from flaxseed oil and the nanoemulsion’s structural properties that promote intimate contact with the fungal cell membrane and facilitate the penetration of hydrophobic molecules into the cell interior. Nevertheless, additional investigations are required to identify the exact antimicrobial mechanisms exerted by the tested nanoemulgel.

In contrast to other studies focusing on either *L. usitatissimum* seed oil or traditional topical formulations, the present work presents a complete characterization of an optimized *L. usitatissimum* seed oil nanoemulgel by merging physicochemical optimization with biological evaluation. The multifunctional nature of the proposed formulation is shown by the favorable droplet size, outstanding colloidal stability, antioxidant activity, elastase inhibition, antibacterial efficacy, and intrinsic sun-protective qualities. These results imply that nanoemulgel technology is a promising approach to enhance the pharmacological and cosmeceutical properties of *L. usitatissimum* seed oil without affecting its biological activity.

## 5. Conclusion

In the present study, a self-nanoemulsifying strategy was employed to successfully formulate an optimized *Linum usitatissimum* oil nanoemulgel, which was incorporated into a Carbopol® gel matrix. The optimized formulation showed good physicochemical properties such as mean droplet size of less than 200 nm, narrow size distribution, good colloidal stability, appropriate rheological behavior and good spreadability for topical application. Moreover, the crude seed oil exhibited excellent intrinsic antioxidant, elastase inhibitory, and photoprotective (SPF) properties, while its incorporation into the optimized nanoemulgel significantly enhanced antimicrobial efficacy against *S. aureus*, MRSA, and *C. albicans* compared to the unformulated oil (p < 0.05). These combined findings highlight the potential of *L. usitatissimum* oil nanoemulgel as a promising candidate for multifunctional topical cosmeceutical applications. Although the good physicochemical features of the nanoemulgel could be responsible for better biological performance, skin permeability or penetration was not directly evaluated in this work. Therefore, further in vitro and ex vivo permeation investigations are needed for the validation of the effect of nanoemulgel.
